# Central pancreatectomy: a Latin American experience of parenchyma-sparing surgery for benign and low-grade pancreatic neoplasms

**DOI:** 10.1590/0102-67202026000003e1932

**Published:** 2026-06-22

**Authors:** Danny CONDE-MONROY, Guilherme Naccache NAMUR, Francisco TUSTUMI, Lucas Cata Preta STOLZEMBURG, Ricardo JUREIDINI, José JUKEMURA, Ulysses RIBEIRO, Paulo HERMAN, Thiago Costa RIBEIRO

**Affiliations:** 1 Universidade de São Paulo, Department of Gastroenterology – São Paulo (SP), Brazil.; 2 Hospital Universitario Mayor-Mederi, Department of Surgery – Bogota, Colombia.

**Keywords:** Pancreas, Pancreatectomy, Pancreatic neoplasms, Pâncreas, Pancreatectomia, Neoplasias pancreáticas

## Abstract

**Background:**

Central pancreatectomy (CP) is a parenchyma-sparing alternative to standard resections for benign or low-grade lesions of the pancreatic neck. While it aims to preserve endocrine and exocrine function, it is associated with significant technical complexity and high rates of postoperative pancreatic fistula (POPF).

**Aims:**

To analyze the CP at a single high-volume Brazilian center.

**Methods:**

A retrospective analysis of a prospectively maintained database was conducted. All patients undergoing CP at a single high-volume Brazilian center between January 2009 and December 2024 were included. Data on demographics, operative details, pathology, complications (International Study Group of Pancreatic Surgery – ISGPS/Clavien-Dindo criteria), and long-term pancreatic function were collected.

**Results:**

Twenty-two patients underwent CP (mean age 54 years, 72% female). The majority of lesions were cystic (50%) or neuroendocrine tumors (36.4%). The POPF rate was 86.4%, all Grade B, most managed conservatively via prolonged drainage. No Grade C fistulas, postoperative hemorrhages, or mortality occurred. Delayed gastric emptying occurred in 22.7%. After a median follow-up of 5.59 years, endocrine insufficiency developed in 9% of patients without prior diabetes (none insulin-dependent), and exocrine insufficiency in 13.6%. Only one locoregional recurrence was observed (isolated metastasis).

**Conclusions:**

This first Latin American series demonstrates that central pancreatectomy is a feasible and effective parenchyma-sparing procedure. It provides excellent long-term preservation of pancreatic function with low severe morbidity, despite high rates of manageable POPF. These outcomes support its role as a valuable surgical option for selected patients in experienced centers.

## ARTICLE HIGHLIGHTS

Central pancreatectomy is a feasible parenchyma-sparing option for benign and low-grade pancreatic neck lesions in experienced centers.

Despite a high rate of postoperative pancreatic fistula, morbidity is predominantly moderate and managed conservatively, with no severe fistulas or mortality.

Long-term endocrine and exocrine pancreatic function is largely preserved, with very low rates of clinically relevant insufficiency.

## Summary

**CENTRAL MESSAGE:**

This original Latin American series demonstrates that central pancreatectomy can be safely performed for selected benign and low-grade pancreatic neoplasms, offering substantial preservation of pancreatic function. Although postoperative pancreatic fistula was frequent, all cases were grade B and predominantly managed without invasive interventions. The absence of severe complications, reoperations, or mortality, combined with excellent long-term endocrine and exocrine outcomes, supports central pancreatectomy as a valuable alternative to standard pancreatic resections when performed in high-volume, experienced centers.

**PERSPECTIVES:**

This study reinforces the long-term functional value of central pancreatectomy. The findings highlight that a high incidence of postoperative pancreatic fistula does not necessarily translate into severe complications or compromised recovery when managed within structured protocols. By demonstrating durable preservation of endocrine and exocrine function with acceptable oncologic control, this experience supports a more nuanced, patient-centered approach to pancreatic surgery, encouraging refinement of indications and perioperative strategies for parenchyma-sparing resections.

## VISUAL ABSTRACT

**Figure:**
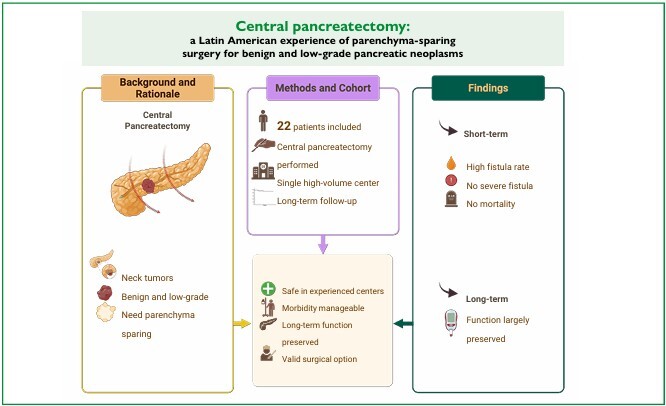

## RESUMO

**Racional:**

A pancreatectomia central (PC) é uma alternativa poupadora de parênquima às ressecções padrão para lesões benignas ou de baixo grau do colo do pâncreas. Embora vise preservar a função endócrina e exócrina, está associada a complexidade técnica significativa e altas taxas de fístula pancreática pós-operatória (FPPO).

**Objetivos:**

Analisar o PC em um único centro brasileiro de alto volume.

**Métodos:**

Foi realizada uma análise retrospectiva de um banco de dados prospectivamente mantido. Todos os pacientes submetidos à PC em um único centro brasileiro de alto volume, entre janeiro de 2009 e dezembro de 2024, foram incluídos. Dados demográficos, detalhes operatórios, patologia, complicações (critérios ISGPS/Clavien-Dindo) e função pancreática de longo prazo foram coletados.

**Resultados:**

Vinte e dois pacientes foram submetidos à PC (idade média de 54 anos, 72% do sexo feminino). A maioria das lesões era cística (50%) ou tumores neuroendócrinos (36,4%). A taxa de FPPO foi de 86,4%, todas do Grau B, sendo a maioria manejada de forma conservadora com drenagem prolongada. Não ocorreram fístulas Grau C, hemorragias pós-operatórias ou mortalidade. O esvaziamento gástrico retardado ocorreu em 22,7%. Após um seguimento mediano de 5,59 anos, a insuficiência endócrina desenvolveuse em 9% dos pacientes sem diabetes prévio (nenhum insulinodependente), e a insuficiência exócrina em 13,6%. Apenas uma recidiva locorregional foi observada (metástase isolada).

**Conclusões:**

Esta primeira série latino-americana demonstra que a pancreatectomia central é um procedimento poupador de parênquima viável e eficaz. Ela proporciona excelente preservação da função pancreática em longo prazo, com baixa morbidade grave, apesar das altas taxas de FPPO manejável. Esses resultados apoiam seu papel como uma opção cirúrgica valiosa para pacientes selecionados em centros experientes.

## INTRODUCTION

 The widespread use of high-resolution abdominal imaging modalities, such as computed tomography and magnetic resonance imaging, has led to a growing detection of incidental pancreatic lesions. While many of these lesions are benign or have low malignant potential, a meaningful proportion still requires surgical resection due to the risk of malignization^7,27^. 

 Pancreatic resections have improved significantly in the past decades^16,18,20^. However, standard pancreatic resections — pancreaticoduodenectomy (PD), distal pancreatectomy (DP), and total pancreatectomy — may result in substantial postoperative functional impairment, particularly when performed for non-malignant disease. Postoperative new-onset diabetes has been reported in 14% of patients after PD and 23% after DP, whereas exocrine insufficiency, presenting with steatorrhea and weight loss, occurs in 44.9 and 17%, respectively^3^. 

 Central pancreatectomy (CP), first described by Dagradi and Serio in 1984, is a parenchyma-sparing procedure indicated for benign or low-grade malignant lesions of the pancreatic neck when enucleation is not feasible^15^. By preserving pancreatic parenchyma, CP aims to minimize postoperative exocrine and endocrine insufficiency compared with conventional resections^15^. The most common indications for CP include low-grade neuroendocrine tumors, non-invasive intraductal papillary mucinous neoplasms (IPMNs), pancreatic cysts, and solid pseudopapillary tumors located in the neck or proximal body^16,17^. 

 Despite its theoretical advantages, CP remains technically demanding and is associated with higher rates of short-term complications, mainly because it requires managing two pancreatic transection sites^11^. 

 Published experience with CP in Latin America remains limited. This study presents outcomes from patients undergoing CP at a single institution in Brazil, aiming to contribute to regional data and to clarify the role of this procedure in clinical practice. 

## METHODS

### Patient selection and study design

 We conducted a retrospective analysis of a prospectively maintained database from the Pancreatic Surgical Unit of the Instituto do Câncer do Estado de São Paulo, Hospital das Clínicas da Faculdade de Medicina da Universidade de São Paulo. All patients who underwent CP between January 2009 and December 2024 were included. 

### Data collection

 Data collected included demographic variables (age, body mass index [BMI]); clinical characteristics (presenting symptoms, American Society of Anesthesiology [ASA] score, preoperative diabetes, and pancreatic exocrine insufficiency [PEI]); pathological diagnosis; operative details; and postoperative complications and mortality. 

 Preoperative assessment included computed tomography (CT) and/or magnetic resonance imaging (MRI) in all cases. Endoscopic ultrasound (EUS) with fine-needle aspiration was selectively performed for histological confirmation. 

 All cases were discussed in a multidisciplinary tumor board involving hepatopancreatobiliary surgeons, radiologists, oncologists, and gastroenterologists. Surgical candidates were restricted to patients with benign or low-grade malignant lesions located in the pancreatic neck or proximal body, for which enucleation was not feasible due to tumor size or proximity (<1 mm) to the main pancreatic duct. 

### Outcomes and definitions

 Postoperative follow-up included systematic evaluation of endocrine and exocrine pancreatic function. Patients were assessed weekly during the early postoperative period and subsequently at 3-, 6-, and 12-month intervals. Only patients with at least 12 months of follow-up were included in the analysis. 

 Postoperative pancreatic fistula (POPF) and delayed gastric emptying (DGE) were defined according to the International Study Group of Pancreatic Surgery (ISGPS) criteria^2,28^, and complication severity was graded according to the Clavien-Dindo classification^8^. 

 Endocrine function was evaluated using glycated hemoglobin (HbA1c) and/or fasting glucose levels to identify new-onset diabetes mellitus (NODM) or deterioration of pre-existing diabetes. Exocrine function was assessed clinically using a structured questionnaire that addressed symptoms of maldigestion (steatorrhea, flatulence, weight loss, or unexplained malnutrition), as fecal elastase testing was not routinely available. Patients receiving pancreatic enzyme replacement therapy (PERT) were classified as having exocrine insufficiency^4^. 

### Surgical technique

 CP was performed after pancreatic mobilization, with proximal transection near the gastroduodenal artery. Transection was achieved using either a stapler or electrocautery, and the proximal stump was closed according to the surgeon’s preference when required. 

 Distal transection was performed with electrocautery, and reconstruction consisted of a Roux-en-Y end-to-side pancreatojejunostomy. In cases of marked distal pancreatic atrophy, anastomosis was omitted. 

 Lymphadenectomy was performed when indicated for low-grade malignant lesions, and at least one intra-abdominal drain was routinely placed. The procedure was performed through either an open or minimally invasive approach (laparoscopic or robotic). A representative case of robotic-assisted CP is demonstrated in Video 1 (https://youtu.be/vbBZPA8bdCs). 

## RESULTS

 Between 2009 and 2024, 22 patients underwent CP. The main patients’ characteristics and outcomes are summarized in Table 1. The mean age was 54 years (40–69), and 16 patients (72%) were female. The mean BMI was 24 kg/m^2^ (22–28), and 21 individuals (95%) were classified as ASA I–II. Four patients (18.2%) had preoperative diabetes mellitus; only one used insulin, and none reported steatorrhea. 

**Table 1:** Main characteristics of the included patients and outcomes.

Characteristic	Value	
**Patients**	**n**	**22**
Age (years)	mean (range)	54 (40–69)
Female sex	n (%)	16 (72.7)
BMI (kg/m²)	mean (range)	24 (22–28)
ASA I–II	n (%)	21 (95.5)
Preoperative diabetes mellitus	n (%)	4 (18.2)
Open approach	n (%)	16 (72.7)
Minimally invasive approach	n (%)	6 (27.3)
Length of stay (days)	median (range)	9 (5–23)
Perioperative mortality	n (%)	0 (0)
Reoperation	n (%)	0 (0)
Postoperative pancreatic fistula grade A	n (%)	0 (0)
Postoperative pancreatic fistula grade B	n (%)	19 (86.4)
Postoperative pancreatic fistula grade C	n (%)	0 (0)
Delayed gastric emptying	n (%)	5 (22.7)
Cystic lesions	n (%)	11 (50.0)
Serous cystadenoma	n	5
Solid pseudopapillary neoplasm	n	3
Mucinous cystadenoma	n	1
IPMN	n	2
IPMN with low-grade dysplasia	n	1
IPMN with high-grade dysplasia (non-invasive)	n	1
Solid tumors	n (%)	11 (50.0)
Neuroendocrine tumors	n	8
Mixed ductal neuroendocrine carcinoma	n	1
Microadenomatosis	n	1
Colorectal metastasis	n	1
Tumor size (cm)	mean (range)	2.59 (0.8–7.5)
PNET<2 cm	n	2
PNET ≥2 cm	n	6
PNET grade 1	n	6
PNET grade 2	n	2
PNET grade 3	n	0

BMI: body mass index; ASA: American Society of Anesthesiology; IPMN: intraductal papillary mucinous neoplasms; PNET: pancreatic neuroendocrine tumor.

 An open approach was performed in 16 cases (72.7%), whereas six procedures were minimally invasive (four laparoscopic and two robotic). The mean operative time was 280 minutes (175–420), with no significant differences across surgical approaches. Mean intraoperative blood loss was 183 mL (50–500), and no patient required intraoperative transfusion. Proximal pancreatic transection was performed with a stapler in 12 procedures (54.5%). In the remaining cases, the pancreatic stump was sutured after transection with an energy device in six patients (27.3%) and with a cold blade in four (18.2%). Pancreatic reconstruction was performed mainly using an invagination technique (n=15), followed by the Blumgart method (n=5). Reconstruction was omitted in two patients due to marked distal pancreatic atrophy. 

 There were no perioperative deaths or reoperations. The median length of stay was nine days (5–23). Gastroparesis occurred in five patients (22%; three type A and 2 type B), all of whom belonged to the cohort that developed postoperative pancreatic fistula. No grade C fistulas were observed. Three patients had no postoperative fistula, whereas 19 (86.4%) developed grade B fistulas. Among the grade B cases, 12 (54%) required only prolonged drainage (>21 days), while seven (31.8%) required an intervention (five readmissions, one treated with antibiotic therapy alone, and one percutaneous drainage). 

 Cystic lesions accounted for half of the cases: five serous cystadenomas, three solid pseudopapillary neoplasms, one mucinous cystadenoma, and two IPMNs (one with low-grade dysplasia and one with high-grade dysplasia, both non-invasive). Among the 11 solid tumors, eight were neuroendocrine tumors, one mixed ductal/neuroendocrine carcinoma, one microadenomatosis, and one isolated colorectal metastasis. The mean tumor size was 2.59 cm (0.8–7.5), and seven patients (31%) had a main pancreatic duct >4 mm. Among pancreatic neuroendocrine tumors (PNETs), two tumors measured<2 cm, and six were >2 cm. The mean number of lymph nodes retrieved in PNET and IPMN cases was 4.7 (0–19). No PNETs were grade 3; six were grade 1, and two were grade 2. 

 After a median follow-up of 5.59 years (mean 9.84), only one locoregional recurrence was observed, occurring in the patient with pancreatic colorectal metastasis. No PNET cases developed recurrence. Exocrine insufficiency occurred in three patients (13.6%), all requiring pancreatic enzyme replacement. Among the 18 patients without preoperative diabetes, two developed postoperative diabetes, neither of whom required insulin. No deterioration in glycemic control was observed in the four patients with pre-existing diabetes. 

## DISCUSSION

 CP remains an uncommon pancreatic resection, accounting for fewer than 5% of procedures even in high-volume centers^6^. In our institution, CP accounted for 2.9% of pancreatic resections performed between 2009 and 2024 (22 of 705 cases), a frequency comparable to that reported by the Verona group, which published only 16 new cases over 12 years^6^. The rarity of CP largely reflects the infrequency of benign or low-grade lesions located in the pancreatic neck and the technical complexity of the operation, which requires management of two pancreatic remnants and consequently carries an increased risk of POPF^21^. This risk is further amplified by the frequent coexistence of classical predisposing factors, including soft pancreatic parenchyma, small duct diameter, and benign pathology^12^. 

 In our cohort, 86.4% of patients developed grade B POPF, a rate higher than those described in series published before 2010 (25–50%^1,9,19,22,25,26^ but consistent with more contemporary studies reporting incidences between 60 and 70%^13,21,23^. This alignment with recent data may partly reflect the impact of the updated ISGPS criteria, which increased diagnostic sensitivity by classifying prolonged drain maintenance as a marker of clinically relevant POPF^2^. 

 At our institution, postoperative management is more conservative regarding drain removal, influenced in part by socioeconomic factors that limit rapid access to healthcare services and restrict the availability of interventional radiology. Within this context, only 30% of patients required any intervention beyond prolonged drain retention (>21 days), and just one patient (4.5%) underwent percutaneous drainage — figures comparable to those reported in other international series^13,21,23^. 

 Postoperative hemorrhage, historically reported as a major cause of reoperation with incidence rates of 5–20%^1,9,13,19,21,22,25,26^, was not observed in our cohort. Gastroparesis occurred in 22% of patients, a rate higher than that described in other series^1,13,23,25^. However, this finding should be interpreted with caution, as gastroparesis is inconsistently reported in CP studies^9,19,22,26^, and its true incidence remains unclear. 

 A recent meta-analysis comparing CP with distal pancreatectomy (DP) demonstrated higher overall morbidity, including increased rates of POPF and hemorrhage after CP. In contrast, no significant differences were observed regarding delayed gastric emptying (p=0.49, p>0.05), surgical site infection (p=0.09, p>0.05), reoperation (p=0.43, p>0.05), or mortality (p=0.18, p>0.05)^10^. Despite these findings, our cohort exhibited a low incidence of severe complications, though this observation must be interpreted cautiously due to the limited sample size. 

 From an oncologic standpoint, a theoretical concern is the potential under-treatment of malignant lesions inadequately characterized preoperatively, either due to positive margins or limited lymphadenectomy. In our cohort, half of the cases involved benign cystic lesions—including two noninvasive IPMNs — which pose no concern regarding oncological radicality. Among solid lesions, and consistent with major published series^1,9,13,19,21,23,25,26^, nearly all cases were PNETs resected with negative margins and without lymph node metastasis. 

 The small number of lymph nodes retrieved represents a limitation of our study, a finding echoed in three other published cohorts, including that of Verona, which reported a median of one lymph node retrieved (range, 1–17). This reflects the fact that lymphadenectomy during CP is, by definition, limited to the hepatic hilum, celiac axis, and proximal splenic artery. Nonetheless, there is no evidence that this limited nodal dissection negatively affects recurrence or overall survival^24^. In our series, the only recurrence observed was an isolated metastasis to the pancreatic surface in a patient previously treated for colon cancer, unrelated to the extent of lymphadenectomy. 

 Regarding pancreatic function, the literature reports new-onset diabetes mellitus (NODM) rates of 14–41% after pancreaticoduodenectomy and 12.6–30% after distal pancreatectomy^3,14^. In our cohort, endocrine insufficiency occurred in 9% of patients and exocrine insufficiency in 13%, values comparable to published rates (2.5–14 and 8–22%, respectively). Recent meta-analyses reinforce that CP better preserves pancreatic function, while being associated with prolonged hospital stay and higher POPF rates^5,24,29^. The only multicenter study assessing endocrine outcomes, conducted by Kato et al., demonstrated significantly lower HbA1c levels at multiple postoperative time points in patients who underwent CP compared with those who underwent DP^17^. Considering the clinical burden, economic impact, and long-term morbidity associated with diabetes, preservation of pancreatic function represents a meaningful advantage of CP. 

 This study has limitations inherent to its retrospective nature and small sample size — a common feature among CP cohorts due to the rarity of the indication. Nonetheless, it represents the first Latin American series of central pancreatectomies, with outcomes comparable to international reports, reinforcing CP as a viable surgical alternative for the treatment of benign and low-grade tumors of the pancreatic body in high-volume pancreatic surgery centers. 

## CONCLUSIONS

 Central pancreatectomy is a feasible parenchyma-sparing option for selected benign and low-grade malignant lesions of the pancreatic neck, offering excellent long-term preservation of pancreatic function. Although associated with high rates of postoperative pancreatic fistula, most cases are managed conservatively, with low severe morbidity. These results support its role as a valuable alternative to standard pancreatic resections in experienced centers. 

## Data Availability

The datasets generated and/or analyzed during the current study are available from the corresponding author upon reasonable request.
